# Letter from the Editor in Chief

**DOI:** 10.19102/icrm.2019.101106

**Published:** 2019-11-15

**Authors:** Moussa Mansour


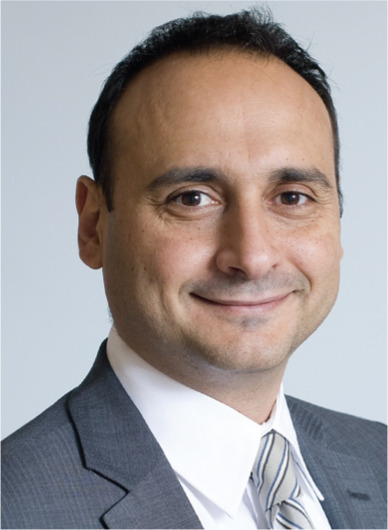


Dear Readers,

Atrial fibrillation (AF) is very prevalent, affecting a large number of people both in the United States and around the world. It has been reported that its incidence has been increasing in part due to the surge in the prevalence rates of obesity and hypertension that we have been observing since the 1980s. It is also known that patients with AF are at a higher risk for death, congestive heart failure, and stroke, making AF a huge clinical and economic burden.

Over the past 20 years, significant advances in the field of cardiac electrophysiology have resulted in improvements in the treatment of AF. Catheter ablation has been shown to enhance the quality of life and survival among patients with AF and congestive heart failure. Studies also indicate that left atrial appendage closure improves survival rates in comparison with warfarin administration in AF patients. While these and other treatments all play an important role in the fight against AF, alone, they remain inadequately effective because of the large number of people affected by this disease. Further, only a relatively small number of patients with AF have the resources to access the more advanced treatments. As a result, taking preventative measures can have an equally important role in fighting AF.

This issue of *The Journal of Innovations in Cardiac Rhythm Management* contains an important article by Drs. Andrade and Macle titled “Addressing Extracardiac Risk Factors to Improve Atrial Fibrillation Treatment Outcomes.”^1^ Here, the authors elegantly describe the different known risk factors for AF including obesity, hypertension, alcohol abuse, and sleep apnea. In particular, not only do they discuss the epidemiological studies linking these conditions to AF but, also, they present the mechanisms of how these risk factors can cause AF and describe strategies to control them, making this article a useful tool for application in clinical practice.

Prevention in the management of AF has been traditionally less emphasized as compared with the prevention of other cardiovascular diseases such as coronary artery disease. Since many of the risk factors for AF are known, developing strategies to control these risk factors is of the utmost importance.

I hope that you enjoy reading this issue and find its content of educational value.

Sincerely,


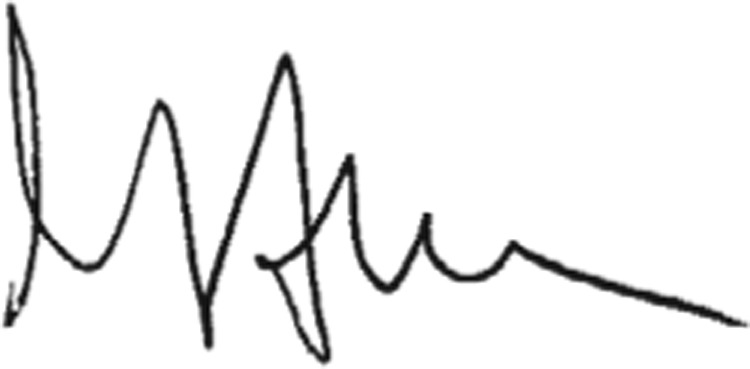


Moussa Mansour, md, fhrs, facc

Editor in Chief

The Journal of Innovations in Cardiac Rhythm Management

MMansour@InnovationsInCRM.com

Director, Atrial Fibrillation Program

Jeremy Ruskin and Dan Starks Endowed Chair in Cardiology

Massachusetts General Hospital

Boston, MA 02114
